# Accounting for the Growth of Observation Stays in the Assessment of Medicare’s Hospital Readmissions Reduction Program

**DOI:** 10.1001/jamanetworkopen.2022.42587

**Published:** 2022-11-17

**Authors:** Amber K. Sabbatini, Karen E. Joynt-Maddox, Josh Liao, Anirban Basu, Canada Parrish, William Kreuter, Brad Wright

**Affiliations:** 1 Department of Emergency Medicine, University of Washington School of Medicine, Seattle; 2 Center for Health Economics and Policy, Institute for Public Health, Washington University in St Louis, St Louis, Missouri; 3 Division of Cardiology, Washington University School of Medicine, St Louis, Missouri; 4 Department of Medicine, University of Washington School of Medicine, Seattle; 5 Value System Science Lab, Department of Medicine, University of Washington, Seattle; 6 The Comparative Health Outcomes, Policy, and Economics Institute, University of Washington School of Pharmacy, Seattle; 7 Department of Health Services, Policy and Management University of South Carolina School of Public Health, Columbia

## Abstract

**Importance:**

Decreases in 30-day readmissions following the implementation of the Medicare Hospital Readmissions Reduction Program (HRRP) have occurred against the backdrop of increasing hospital observation stay use, yet observation stays are not captured in readmission measures.

**Objective:**

To examine whether the HRRP was associated with decreases in 30-day readmissions after accounting for observation stays.

**Design, Setting, and Participants:**

This retrospective cohort study included a 20% sample of inpatient admissions and observation stays among Medicare fee-for-service beneficiaries from January 1, 2009, to December 31, 2015. Data analysis was performed from November 2021 to June 2022. A differences-in-differences analysis assessed changes in 30-day readmissions after the announcement of the HRRP and implementation of penalties for target conditions (heart failure, acute myocardial infarction, and pneumonia) vs nontarget conditions under scenarios that excluded and included observation stays.

**Main Outcomes and Measures:**

Thirty-day inpatient admissions and observation stays.

**Results:**

The study included 8 944 295 hospitalizations (mean [SD] age, 78.7 [8.2] years; 58.6% were female; 1.3% Asian; 10.0% Black; 2.0% Hispanic; 0.5% North American Native; 85.0% White; and 1.2% other or unknown). Observation stays increased from 2.3% to 4.4% (91.3% relative increase) of index hospitalizations among target conditions and 14.1% to 21.3% (51.1% relative increase) of index hospitalizations for nontarget conditions. Readmission rates decreased significantly after the announcement of the HRRP and returned to baseline by the time penalties were implemented for both target and nontarget conditions regardless of whether observation stays were included. When only inpatient hospitalizations were counted, decreasing readmissions accrued into a −1.48 percentage point (95% CI, −1.65 to −1.31 percentage points) absolute reduction in readmission rates by the postpenalty period for target conditions and −1.13 percentage point (95% CI, −1.30 to −0.96 percentage points) absolute reduction in readmission rates by the postpenalty period for nontarget conditions. This reduction corresponded to a statistically significant differential change of −0.35 percentage points (95% CI, −0.59 to −0.11 percentage points). Accounting for observation stays more than halved the absolute decrease in readmission rates for target conditions (−0.66 percentage points; 95% CI, −0.83 to –0.49 percentage points). Nontarget conditions showed an overall greater decrease during the same period (−0.76 percentage points; 95% CI, −0.92 to −0.59 percentage points), corresponding to a differential change in readmission rates of 0.10 percentage points (95% CI, −0.14 to 0.33 percentage points) that was not statistically significant.

**Conclusions and Relevance:**

The findings of this study suggest that the reduction of readmissions associated with the implementation of the HRRP was smaller than originally reported. More than half of the decrease in readmissions for target conditions appears to be attributable to the reclassification of inpatient admission to observation stays.

## Key Points

**Question:**

To what extent does the increase of observation stays account for the decrease in readmissions associated with the Medicare Hospital Readmissions Reduction Program (HRRP)?

**Findings:**

In this cohort study including 8 944 295 hospitalizations, fully accounting for observation stays as both index hospital discharges and readmissions more than halved the apparent decrease in 30-day readmissions (−1.48 vs −0.66 percentage points). In addition, an association of the program with lower readmission rates identified when only inpatient hospitalizations were considered was not found.

**Meaning:**

The findings of this study suggest that much of the estimated reduction in readmissions associated with the implementation of the HRRP can be attributed to reclassification of inpatient admissions to observation stays.

## Teaser

This cohort study examines the association between the Hospital Readmissions Reduction Program and 30-day readmissions after accounting for observation stays.

## Introduction

The Hospital Readmissions Reduction Program (HRRP), legislated as part of the Patient Protection and Affordable Care Act (ACA), levies financial penalties on hospitals with higher-than-expected readmission rates for certain conditions. Several studies have suggested that the implementation of the HRRP was associated with modest decreases in readmissions shortly after the program was announced in 2010, especially for conditions and hospitals targeted by the program.^1,2,3,4,5,6,7,8^

However, decreasing readmission rates have occurred against the backdrop of increasing hospital observation stay use. Medicare policies overlapping the implementation of the HRRP, including the Recovery Audit Contractor program^9,10^ (which led to payment denials for short inpatient admissions) and the 2-Midnight Rule^11,12^ (which advised inpatient admission was generally inappropriate for hospitalizations crossing fewer than 2 midnights), expanded the use of observation by hospitals and resulted in many inpatient admissions being reclassified as observation stays.^13,14^ Consequently, observation stays increased sharply in the run-up to the ACA^15^ and continued to increase through the period of HRRP implementation,^16^ such that approximately 18% of Medicare beneficiaries now complete their hospital treatment in observation.^17^ In many cases, these observation stays are clinically indistinguishable from short inpatient admissions with patients hospitalized for observation sharing the same clinical wards and teams as inpatients.

The rapid growth of observation stays makes understanding the association between the HRRP and hospital readmissions challenging given that observation stays are not counted as index hospitalizations or readmissions in the calculation of readmission rates. Because HRRP evaluations rely on longitudinal study designs, increased use of observation stays over time could thus lead to an overestimation of HRRP outcomes. Some descriptive studies suggest that most of the decreases in total readmissions since passage of the ACA can be explained by the reclassification of inpatient readmissions to observation stays.^18,19,20^ Other studies that have examined the association between the HRRP and observation stays use have considered observation stays in the postdischarge period^2,21^ but have not accounted for observation stays that have increasingly substituted for index inpatient hospitalizations.

Therefore, the goal of this study was to reexamine whether the HRRP was associated with reductions in readmissions when fully accounting for observation stays as both index hospital discharges and readmissions. We also sought to evaluate whether there was any spillover between the HRRP and rehospitalization for Medicare enrollees with observation stays, in terms of reducing rehospitalization following an index observation visit.

## Methods

The University of Washington Human Subjects Division approved this study and determined it qualified for a waiver of consent due to the use of deidentified data. This study adheres to the Strengthening the Reporting of Observational Studies in Epidemiology (STROBE) reporting guideline for cohort studies.

### Data Source and Study Population

We used Medicare Part A and B claims to generate a 20% sample of inpatient admissions and observation stays at short-term hospitals occurring between January 1, 2008, and December 31, 2015. Observation stays were identified from outpatient claims with a revenue center code of 0760 or 0762. We classified observation stays that subsequently converted to inpatient status as inpatient discharges for outcomes assessment. We used the same inclusion and exclusion criteria as the Centers for Medicare & Medicaid (CMS) Hospital Wide Readmission measure to define this sample.^22^ We excluded hospitalizations in Maryland and critical access hospitals because these were exempt from the HRRP.

Index hospitalizations were sorted into 2 groups based on principal diagnosis using Clinical Classifications Software (CCS) from the Agency for Healthcare Research and Quality^23^: a combined group of 3 conditions initially targeted by the HRRP (acute myocardial infarction, heart failure, and pneumonia) and a comparator group of remaining conditions not targeted by the HRRP. To select the most appropriate comparator group, we first examined whether each nontarget CCS diagnosis group exhibited similar prepolicy parallel trends in readmissions to the combined target group, excluding 18 CCS conditions (n = 2 317 874) with nonparallel trends from the final nontarget group (eTable 1 in the Supplement). We also omitted hospitalizations for chronic obstructive pulmonary disease and hip and knee replacement because these conditions were targeted by the HRRP later.

### Outcomes

We examined 30-day readmissions under different scenarios. In our base scenario, we assessed changes in inpatient readmissions within 30 days of inpatient discharges only, which is the current CMS definition of a readmission. In an expanded scenario, we assessed changes in inpatient readmissions when observations were considered both as index hospitalizations (denominator of readmission measure) and as 30-day readmissions (numerator of readmission measure), comparing results between the base and expanded scenarios. We used the CMS unplanned readmission algorithm^22^ to exclude readmissions that were likely to be planned, such as staged coronary intervention or chemotherapy.

### Statistical Analysis

Data analysis was conducted from November 2011 to June 2022. We used a difference-in-differences (DID) approach to assess the outcomes of the HRRP, comparing changes in 30-day readmissions across 3 periods: a baseline period before ACA passage (January 1, 2009, to March 31, 2010), an intervening period after ACA passage and HRRP announcement but before penalty implementation (April 1, 2010, to September 30, 2012), and a postpenalty period (October 1, 2012, to December 31, 2015), replicating methods in earlier studies.^3,8,24^ This process entailed a 2-stage approach wherein we first generated a single risk-adjusted monthly readmission rate for each of the combined target and nontarget groups using logistic regression. Models adjusted for age, sex, 31 comorbidity groups, principal discharge diagnosis, and hospital fixed effects. Data on race and ethnicity are given as a demographic characteristic of the treatment groups; the information was not used for risk adjustment in readmission rates. We limited capture of comorbidities to the first 9 diagnoses on claims records to avoid bias from Medicare coding changes that occurred during the study period.^24,25^ In the second stage, we used linear probability DID models that incorporated indicators for target group, policy period, and continuous month, and interactions between the 3 indicators to estimate the association between the HRRP and changes in risk-adjusted readmission rates estimated from our first-stage model under both scenarios. Analyses were conducted with SAS, version 9.4 (SAS Institute Inc), and Stata version 16 (StataCorp LLC). All tests for statistical significance were 2-tailed and evaluated at a significance level of *P* < .05.

We performed multiple sensitivity analyses around our control group. First, the population of observation stays include a preponderance of lower acuity, symptom-based hospitalizations, such as syncope or chest pain, that may not be comparable to the conditions targeted by the HRRP. Thus, we restricted our nontarget group to conditions in which observation stays comprised less than 10% of total hospitalizations, thereby comparing high-acuity target conditions with a similar group of nontarget conditions predominantly managed as inpatients. Second, given concerns about spillover of the HRRP into the nontarget group, we also compared changes in readmissions for target conditions at HRRP-exposed hospitals vs hospitals that were exempt from the HRRP (eg, critical access, Maryland, and federal hospitals) in separate DID analysis.

## Results

### Characteristics of the Study Population

After exclusions, our study included 8 944 295 index hospitalizations (mean [SD] age, 78.7 [8.2] years; 58.6% were female; 1.3% Asian; 10.0% Black; 2.0% Hispanic; 0.5% North American Native; 85.0% White; and 1.2% other or unknown) (eFigure and eTable 2 in the Supplement). Of these, 1 406 451 (15.7%) were for 1 of the 3 conditions initially targeted by the HRRP. For the overall study cohort, patients hospitalized for target conditions were more likely to be older (mean [SD] age, 79.9 [8.3] vs 78.5 [8.1] years) and have more comorbidities (mean [SD] 6.9 [3.0] vs 5.6 [3.3] conditions) with a greater proportion of men (46.2% vs 40.5%) compared with those with nontarget conditions (eTable 2 in the Supplement). Only 3.3% of hospital discharges among target conditions were observation stays compared with 17.9% of nontarget conditions.

During the study period, the number of men in the population, as well as the number of comorbidities, increased, with fewer patients dually enrolled in Medicaid (Table 1). These demographic shifts were similar for target and nontarget conditions. In addition, observation stays increased throughout the study period. Among target conditions, the proportion of total hospitalizations that were observation stays increased from 2.3% in the baseline period to 4.4% in the postpenalty period (91.3% relative increase). Among nontarget conditions, observation stays increased from 14.1% to 21.3% of total hospitalizations during the same periods (51.1% relative increase).

**Table 1.** Characteristics of Target and Nontarget Hospitalizations Over Time

Characteristic	Baseline		HRRP announced		HRRP penalties implemented	
	Target conditions (n = 436 532)	Nontarget conditions (n = 2 184 490)	Target conditions (n = 437 670)	Nontarget conditions (n = 2 382 788)	Target conditions (n = 532 249)	Nontarget conditions (n = 2 970 566)
Age, mean (SD), y	79.9 (8.1)	78.5 (8.0)	80.0 (8.2)	78.5 (8.1)	79.8 (8.4)	78.4 (8.3)
Sex, %						
Women	54.5	59.9	54.0	59.9	53.0	58.9
Men	45.5	40.1	46.0	40.1	47.0	41.1
Race and ethnicity, %						
Asian	1.2	1.2	1.3	1.3	1.4	1.4
Black	9.3	10.0	9.7	10.3	9.6	10.1
Hispanic	2.0	2.1	2.0	2.1	1.9	1.9
North American Native	0.5	0.4	0.5	0.5	0.5	0.5
White	86.1	85.4	85.6	84.8	85.4	84.6
Other	0.8	0.9	0.9	1.0	1.0	1.1
Unknown	0.1	0.1	0.1	0.1	0.3	0.4
Dual eligibility status, %	26.0	25.6	25.6	25.2	23.7	23.5
Comorbidities, mean (SD)	6.8 (3.0)	5.4 (3.3)	7.0 (3.0)	5.6 (3.3)	7.0 (3.0)	5.7 (3.3)
Type of hospitalization, %						
Inpatient	97.8	85.9	97.1	82.9	95.6	78.7
Observation stay	2.3	14.1	2.9	17.1	4.4	21.3
Length of stay, mean (SD), d	6.2 (4.3)	5.4 (4.7)	6.0 (4.3)	5.2 (4.6)	5.7 (4.0)	5.1 (4.6)
Rehospitalization at 30 d, %	23.6	18.5	23.3	18.4	22.3	17.9
Inpatient discharge	22.3	17.0	21.7	16.7	20.1	15.7
Observation stay discharge	1.7	1.8	2.2	2.2	2.9	2.8

Abbreviation: HRRP, Hospital Readmission Reduction Program.

### Trends in Rehospitalization

The Figure and eTable 3 in the Supplement present the adjusted trends (slope) in 30-day readmissions for target and nontarget conditions under our base and expanded scenarios. Similar to earlier work, we found that readmissions for target conditions decreased at a faster rate in the period after the announcement of the HRRP (mean change in slope, −0.41 percentage points per year; 95% CI, −0.64 to −0.18 percentage points) but returned to baseline in the postpenalty period. Accounting for observation stays found these same trends (mean change in slope, −0.39 percentage points per year; 95% CI, −0.63 to −0.15 percentage points). Under both scenarios, trends in readmissions also decreased for nontarget conditions, although less than for target conditions.

**Figure.**
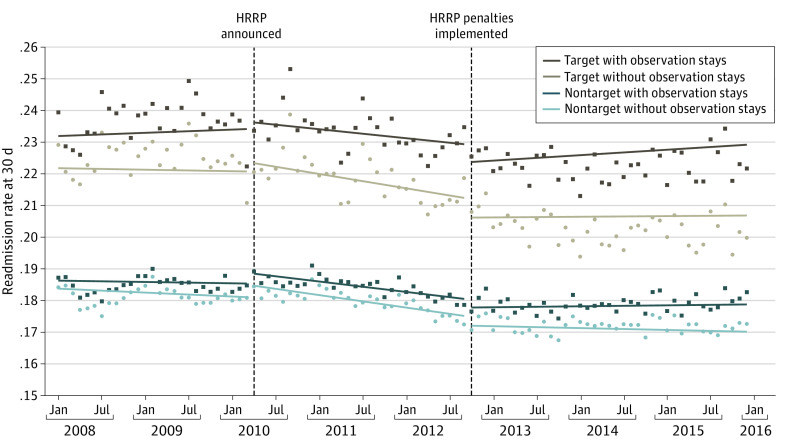
Changes in Target vs Nontarget Observation Stays in 30-Day Readmissions Before and After Implementation of the Medicare Hospital Readmissions Reduction Program (HRRP) Points represent monthly adjusted readmission rates; solid lines represent the fitted trend from regression models.

### Estimated Outcomes After Accounting for Observation Stays

Table 2 reports the association of the HRRP with risk-adjusted readmission rates from our DID analysis. Under the base scenario without observation stays, the combined readmission rate for target conditions decreased progressively from a mean of 22.14% in the baseline period to 20.65% in the post-HRRP penalty period (6.7% relative decrease; difference, −1.48 percentage points; 95% CI, −1.65 to −1.31 percentage points). For nontarget conditions, the readmission rate decreased from a mean of 18.24% to 17.11% (6.2% relative decrease; difference, −1.13 percentage points; 95% CI, −1.30 to −0.96 percentage points). This decrease was estimated to be a small, but significant, differential change in readmissions for target conditions compared with nontarget conditions of −0.35 percentage points (95% CI, −0.59 to −0.11 percentage points) in the post-HRRP penalty period.

**Table 2.** Estimated Associations of the HRRP With Risk-Adjusted Rate of 30-Day Readmissions Under Scenarios With and Without Observation Stays

Characteristic	% (95% CI)				
	Baseline, period 1	HRRP announced, period 2	Change, period 2 − period 1	HRRP penalties implemented, period 3	Change, period 3 − period 1
Base scenario (inpatient readmissions following inpatient discharges only)					
Target	22.14 (22.00 to 22.27)	21.80 (21.67 to 21.92)	−0.34 (−0.52 to −0.16)	20.65 (20.55 to 20.76)	−1.48 (−1.65 to −1.31)
Nontarget	18.24 (18.11 to 18.37)	17.99 (17.86 to 18.11)	−0.25 (−0.43 to −0.07)	17.11 (17.00 to 17.21)	−1.13 (−1.30 to −0.96)
Difference-in-differences	NA	NA	−0.09 (−0.35 to 0.17)	NA	−0.35 (−0.59 to −0.11)
Expanded scenario (observation stays counted as index discharges and readmission events)					
Target	23.32 (23.08 to 24.15)	23.29 (23.01 to 23.28)	−0.03 (−0.20 to 0.15)	22.66 (21.98 to 22.51)	−0.66 (−0.83 to −0.49)
Nontarget	18.58 (18.45 to 18.71)	18.45 (18.33 to 18.57)	−0.13 (−0.31 to 0.47)	17.82 (17.72 to 17.93)	−0.76 (−0.92 to −0.59)
Difference-in-differences	NA	NA	0.11 (−0.14 to 0.36)	NA	0.10 (−0.14 to 0.33)

Abbreviations: HRRP, Hospital Readmission Reduction Program; NA, not applicable.

In the expanded scenario accounting for observation stays, the absolute reduction in readmission rate for target conditions was more than halved, decreasing from 23.32% in the baseline period to 22.66% in the post-HRRP penalty period (2.8% relative decrease; difference, −0.66 percentage points; 95% CI, −0.83 to –0.49 percentage points). In addition, nontarget conditions showed a slightly larger absolute decrease from a rate of 18.58% to 17.82% (4.1% relative decrease; difference, −0.76 percentage points; 95% CI, −0.92 to −0.59 percentage points), which corresponded to an nonsignificant differential change of 0.10 percentage points (95% CI, −0.14 to 0.33 percentage points) in the postpenalty period.

To examine our secondary question of whether there was any spillover of the HRRP on rehospitalizations for Medicare beneficiaries with observation stays, we examined changes in any unplanned rehospitalization stratified by type of index hospital discharge (Table 3). First, for inpatient discharges, modification of the definition of a readmission to include either inpatient admissions or observation stays at 30 days (counting observation stays in the numerator of the readmission outcome only) resulted in findings similar to our expanded scenario that included observation stays in both the numerator and denominator. For observation discharges among target conditions, rates of rehospitalization increased slightly after the announcement of the HRRP but ultimately decreased by 0.48 percentage points (95% CI, −0.83 to −0.14 percentage points) in the post-HRRP period vs a 0.72 percentage point decrease (95% CI, −0.89 to −0.56 percentage points) for inpatient discharges. Although rehospitalizations for observation discharges in the nontarget group showed small decreases over time, these changes were not found to be significantly different from baseline.

**Table 3.** Estimated Associations of the HRRP With Any Unplanned Rehospitalization at 30 Days, Stratified by Type of Index Discharge

Characteristic	% (95% CI)				
	Baseline, period 1	HRRP announced, period 2	Change, period 2 − period 1	HRRP penalties implemented, period 3	Change, period 3 − period 1
Inpatient discharges					
Target	23.44 (23.32 to 23.57)	23.40 (23.28 to 23.52)	−0.04 (−0.21 to −0.13)	22.72 (22.62 to 22.82)	−0.72 (−0.89 to −0.56)
Nontarget	19.37 (19.25 to 19.50)	19.42 (19.30 to 19.53)	0.05 (−0.13 to 0.22)	18.95 (18.85 to 19.06)	−0.42 (−0.58 to −0.25)
Difference-in-differences	NA	NA	−0.09 (−0.33 to 0.16)	NA	−0.31 (−0.54 to −0.79)
Observation stay discharges					
Target	20.18 (19.91 to 20.45)	20.36 (20.11 to 20.62)	0.19 (−0.18 to 0.55)	19.69 (19.47 to 19.92)	−0.48 (−0.83 to −0.14)
Nontarget	13.82 (13.55 to 14.09)	13.72 (13.47 to 13.98)	−0.10 (−0.46 to 0.27)	13.70 (13.48 to 13.93)	−0.12 (−0.46 to 0.23)
Difference-in-differences	NA	NA	0.28 (−0.24 to 0.80)	NA	−0.36 (−0.86 to 0.12)

Abbreviations: HRRP, Hospital Readmission Reduction Program; NA, not applicable.

Our sensitivity analysis restricting the control group to the subset of nontarget conditions with observation stays comprising less than 10% of total hospitalizations yielded nearly identical results to our main analysis. In our sensitivity analysis comparing changes in readmissions for target conditions at HRRP-exposed and HRRP-exempt hospitals, we found greater decreases at HRRP-exempt hospitals in risk-adjusted readmissions over time compared with HRRP-exposed hospitals, regardless of whether observation stays were included in the calculation of rates (eTable 4 in the Supplement). This decrease resulted in a positive significant differential change of 0.49 percentage points in models that excluded observation stays and 0.50 percentage points in models that included observation stays. Observation stays explained approximately 40% of the change in rates over time.

## Discussion

In this study, we accounted for growth of hospital observation stays to examine the association between the HRRP and 30-day readmissions. Similar to prior work,^2^ we found that readmissions decreased at a significantly faster rate after the announcement of the HRRP for both target and nontarget conditions, with trends returning to baseline by the time penalties were implemented. When only inpatient hospitalizations were considered, implementation of the HRRP was associated with a small but statistically significant decrease in the rate of readmissions for target conditions compared with nontarget conditions of 0.35 percentage points (or approximately 40 000 fewer hospitalizations per year). Hospital observation stays doubled for target conditions after HRRP implementation, yet these observation stays remained a small fraction of index hospitalizations (<5%) and 30-day rehospitalization events (<3%). Nonetheless, accounting for observation stays halved the apparent reduction in readmission rates and negated the significant DID estimate previously identified in our inpatient-only analysis.

Our study fills a gap in the HRRP literature in that we fully account for the increase of observation stays in both the numerator and denominator of readmission rates in the assessment of HRRP outcomes. There have been long-standing concerns that hospitals may attempt to avoid readmission penalties by placing patients in observation during a readmission. To address this concern, earlier work has largely focused on whether the HRRP increased rates of postdischarge observation use (counted observation in the numerator only) and has modeled observation use separately from inpatient readmissions. Zuckerman et al^2^ found that, although observation stays increased steadily throughout HRRP implementation, there was no significant change in the rate (slope) of 30-day observation use, as well as no correlation between postdischarge observation use and decreases in inpatient readmissions within hospitals. Other investigators have found that certain groups of hospitals may have increased observation stays,^26^ but the increase can explain only a small portion of the reduction in readmissions.^21,27,28^

However, trends in observation stays are important beyond the question of whether hospitals have attempted to game the program in any systematic way. Ignoring the growth of observation stays results in a measurement problem for estimating the potential outcomes associated with HRRP. Observation stays replaced a substantial portion of index admissions during HRRP implementation. Readmissions associated with these index events—nearly 1 in 5 hospitalizations in the Medicare population^29^—have fallen out of the calculation of readmission rates over time in a nonrandom way, introducing bias in longitudinal assessments of the HRRP to date, as well as misclassifying the true performance of hospitals. Our results suggest that an increasingly larger share of hospital care will be invisible to quality metrics if shifts in observation stay practices are not accounted for in readmissions algorithms. The resulting risks of incorrect assumptions and program ineffectiveness extend beyond the HRRP to other quality programs, particularly given broader trends to both measure readmissions under value-based payment models and shift more conditions and procedures to outpatient management.

Findings from this study underscore work suggesting that readmissions after implementation of HRRP have decreased less than originally reported. Multiple studies have now reported that upwards of half to two-thirds of the decrease in readmissions following the announcement of the program (the only period during which there is a measurable association between the HRRP and readmissions) are due to statistical bias arising from coding changes that occurred during implementation of the HRRP.^24,25^ In 2011, the CMS increased the number of reportable diagnoses on claims forms from 9 to 25, making the pool of hospitalizations in later years artificially appear to have more severe illness in risk-adjustment models. In our study, we accounted for these coding changes and yet identified further reductions in the potential association between the HRRP and readmissions with the inclusion of observation stays. Other studies have similarly suggested that the significant decrease in readmissions early on may simply reflect regression to the mean^30^ or mirror decreases in inpatient admissions more broadly.^31^ Coupled with a growing body of evidence noting equity concerns about the HRRP^32,33,34,35,36^ and the potential for increases in mortality, at least among patients with heart failure,^31,37,38,39,40,41^ our findings suggest that the program may be underperforming relative to the penalties levied on 93% of hospitals since the inception of the program.^42^

Most readmissions are associated with factors outside the hospital, including social support, access to outpatient care, and social determinants of health.^32,43,44,45^ Observation stays, which are usually short and may occur in an observation unit rather than a typical hospital ward, may not afford the same opportunity for consultation with social work, care coordination, and other key team members. There may also be differences in care protocols between observation and inpatient admissions, as well as incentives to discharge patients from the hospital more quickly if treated in observation status. In our stratified analysis, we noted that readmissions for observation discharges also decreased by the postpenalty period for target conditions (although to less of a degree than for inpatients) while readmissions following observation discharges for nontarget conditions were not significantly different from baseline, perhaps suggesting some modest spillover into the observation population.

However, our findings reinforce the caution needed when evaluating the overall outcomes of the HRRP. Early decreases in readmissions among nontarget conditions have largely been attributed to positive spillover, but may have root causes beyond the HRRP.^46^ Readmissions began decreasing in 2010, before HRRP penalties and before clearly articulated program regulation. Furthermore, decreases in readmissions have been observed in almost every comparator group studied, including hospitals not participating in the HRRP and patients insured through other payers.^8,47^ Attributing readmissions reductions to immediate sweeping effects of a program that spilled over into virtually every patient population is a strong assumption.

A more plausible interpretation is that observed decreases in readmissions reflect secular trends arising from a complex set of factors, including advances in clinical care delivery that reduce the need for inpatient admission, greater use of home health care, better diagnostic tests, and more observation stays. This possibility is further supported by our sensitivity analysis noting that HRRP-exempt hospitals had greater decreases in readmissions over time compared with HRRP-exposed hospitals, as well as work reporting that readmissions in Canada decreased to a similar degree as those in the US following the passage of the ACA, despite not having a program like the HRRP.^48^ If we interpret changes in readmissions for nontarget conditions as even partially reflecting secular trends, then the null DID estimate after including observation stays estimated in our primary analysis suggests that the HRRP may not have been particularly effective.

### Limitations

Our study has limitations. First, our study was not able to identify the extent to which changes in readmissions in nontarget conditions reflected spillover of the HRRP or secular trends. However, the within-group changes in this study were largely consistent with earlier findings. Second, Medicare claims do not differentiate whether observation stays occur in protocol-driven units, an emergency department bed, or the postacute care unit that are qualitatively different than an admission. Third, as is the case with all the evaluations of the HRRP, there were many concurrent policies, programs, and delivery changes implemented during the study period that may have also been associated with readmission rates. Fourth, we did not assess changes in emergency department use in the postdischarge period but acknowledge that increased rates of treat-and-release emergency department visits may have also explained some of the reductions in readmissions attributed to the HRRP.^49^

## Conclusions

Although the announcement of the HRRP was associated with transient decreases in readmission rates, the findings of this cohort study suggest that the outcomes of HRRP regarding readmissions have been less than originally reported. We noted that more than half of the absolute decreases in readmission rates for target conditions was attributable to observation stays.

## Notes

Supplement. **eFigure.** Derivation of Study Sample **eTable 1.** CCS Conditions Excluded From Nontarget Group Due to Nonparallel Baseline Trends **eTable 2.** Characteristics of the Study Population Overall **eTable 3.** Adjusted Trends (Slope) in Rehospitalization Under Scenarios That Exclude and Include Observation Stays **eTable 4.** Sensitivity Analysis Comparing Differential Changes in Readmission Rate Over Time Among HRRP-Exposed and HRRP-Exempt Hospitals, Under Scenarios That Exclude and Include Observation Stays
