# Error in Author’s Name in Byline

**DOI:** 10.1001/jamanetworkopen.2022.51415

**Published:** 2022-12-21

In the Original Investigation titled “Accounting for the Growth of Observation Stays in the Assessment of Medicare’s Hospital Readmissions Reduction Program,”^1^ published November 17, 2022, Dr Liao’s name had been truncated as Josh Liao in the byline; this has been corrected to his full name, Joshua M. Liao. This article has been correted.^1^
