# Supplementary material for: Accounting for the Growth of Observation Stays in the Assessment of Medicare’s Hospital Readmissions Reduction Program
Source: JAMA Netw Open. 2022 Nov 17;5(11):e2242587. doi: 10.1001/jamanetworkopen.2022.42587 (PMC9672971; doi:10.1001/jamanetworkopen.2022.42587)
Supplement: Supplement. — eFigure. Derivation of Study Sample eTable 1. CCS Conditions Excluded From Nontarget Group Due to Nonparallel Baseline Trends eTable 2. Characteristics of the Study Population Overall eTable 3. Adjusted Trends (Slope) in Rehospitalization Under Scenarios That Exclude and Include Observation Stays eTable 4. Sensitivity Analysis Comparing Differential Changes in Readmission Rate Over Time Among HRRP-Exposed and HRRP-Exempt Hospitals, Under Scenarios That Exclude and Include Observation Stays [file jamanetwopen-e2242587-s001.pdf]

Supplementary Online Content

Sabbatini AK, Joynt-Maddox KE, Liao J, et al. Accounting for the growth of observation stays in the assessment of Medicare's Hospital Readmissions Reduction Program. *JAMA Netw Open*. 2022;5(11):e2242587. doi:10.1001/jamanetworkopen.2022.42587

eFigure. Derivation of Study Sample

eTable 1. CCS Conditions Excluded From Nontarget Group Due to Nonparallel Baseline Trends

eTable 2. Characteristics of the Study Population Overall

eTable 3. Adjusted Trends (Slope) in Rehospitalization Under Scenarios That Exclude and Include Observation Stays

eTable 4. Sensitivity Analysis Comparing Differential Changes in Readmission Rate Over Time Among HRRP-Exposed and HRRP-Exempt Hospitals, Under Scenarios That Exclude and Include Observation Stays

This supplementary material has been provided by the authors to give readers additional information about their work.

© 2022 Sabbatini AK et al. *JAMA Network Open.*

eFigure. Derivation of study sample

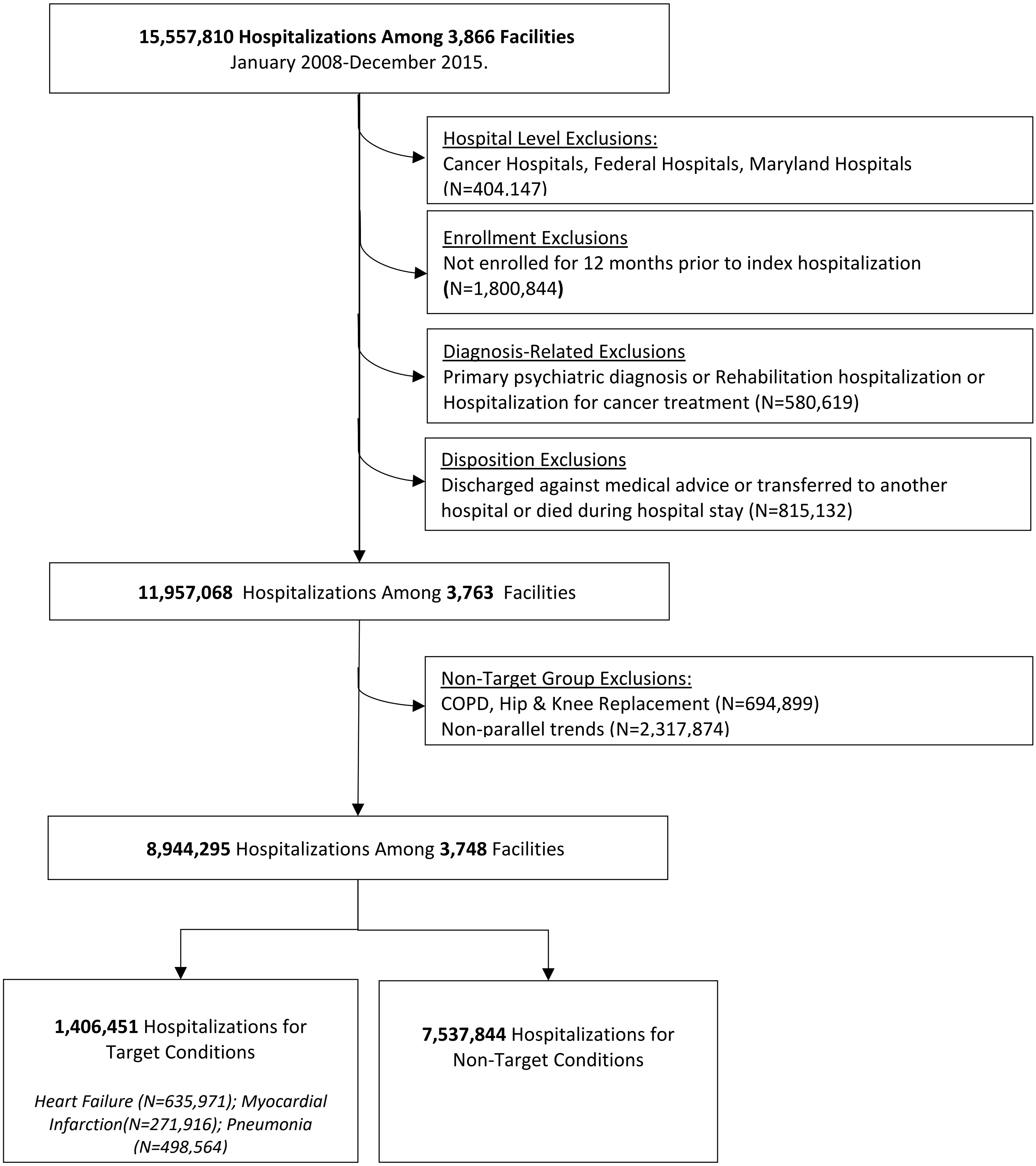

© 2022 Sabbatini AK et al. *JAMA Network Open.*

eTable 1. CCS conditions excluded from nontarget group due to nonparallel baseline trends

Category	N	% total
46 (Benign Uterine Neoplasm)	1,920	0.02
52 (Nutritional deficiencies)	4,340	0.04
76 (Meningitis)	2,323	0.02
106 (Cardiac dysrhythmias)	489,595	4.61
115 (Aortic; peripheral; visceral artery aneurysms)	44,165	0.42
137 (Diseases of mouth; excluding dental)	5,903	0.06
143 (Abdominal hernia)	78,083	0.74
147 (Anal and rectal conditions)	15,166	0.14
149 (Biliary tract disease)	128,977	1.22
157 (Acute and unspecified renal failure)	246,432	2.32
160 (Calculus of urinary tract)	40,668	0.38
162 (Other diseases of bladder and urethra)	11,699	0.11
170 (Prolapse of female genital organs)	32,807	0.31
203 (Osteoarthritis)	447,895	4.22
205 (Spondylosis; back problems)	206,939	1.95
211 (Other connective tissue disease)	68,195	0.64
244 (Other injuries/conditions due to external causes)	31,957	0.30
245 (Other injuries/conditions due to external causes)	205,088	1.93

© 2022 Sabbatini AK et al. *JAMA Network Open.*

eTable 2. Characteristics of the Study Population Overall.

	Overall<br><br>N= 8,944,295	Target Conditions<br>N= 1,406,451	Non-Target Conditions<br>N= 7,537,844
Age, y, mean (SD)	78.7 (8.2)	79.9 (8.3)	78.5 (8.1)
Female, %	5,168,527 (58.6)	743,852 (53.8)	4,424,675 (59.5)
Race/Ethnicity, %			
White	7,601,871 (85.0)	1,204,607 (85.7)	6,397,264 (84.9)
Black	896,193 (10.0)	134,073 (9.5)	762,120 (10.1)
Asian	117,102 (1.3)	17,784 (1.3)	99,318 (1.3)
Hispanic	180,064 (2.0)	27,525 (2.0)	152,539 (2.0)
North American Native	42,364 (0.5)	7,174 (0.5)	35,190 (0.5)
Other	88,309 (1.0)	12,676 (0.9)	75,633 (1.0)
Unknown	18,392 (0.2)	2,612 (0.2)	15,780 (0.2)
Dual Eligibility Status, %	2,210,817 (24.7)	351,626 (25.0)	1,859,191 (24.6)
No. Comorbidities, mean (SD)	5.8 (3.3)	6.9 (3.0)	5.6 (3.3)
Type of Hospitalization, %			
Inpatient	7,550,738 (84.4)	1,360,164 (96.7)	6,190,574 (821)
Observation Stay	1,393,557 (15.6)	46,287 (3.3)	1,347,270 (17.9)
Length of Stay, mean (SD)	5.2 (4.5)	6.0 (4.3)	5.1 (4.5)
Rehospitalization at 30-Days, %			
Inpatient	1,534,392 (17.2)	299,098 (21.3)	1,235,294 (16.4)
Observation Stay	207,092 (2.3)	32,546 (2.3)	174,546 (2.3)

© 2022 Sabbatini AK et al. *JAMA Network Open.*

eTable 3. Adjusted trends (slope) in rehospitalization under scenarios that exclude and include observation stays.

30-Day Event	*Population*	*Group*	Pre-ACA (Baseline)	Post-ACA/Pre-HRRP (Period 2)	Δ (Period 2-Baseline)	Post-HRRP (Period 3)	Δ (Period 3-Baseline)
Inpatient Readmissions	Inpatient Discharges	Target	-0.05 (-0.22, 0.13)	-0.46 (-0.61, -0.30)	-0.41 (-0.64, -0.18)	0.02 (-0.08, 0.12)	0.07 (-0.14, 0.27)
		Non-Target	-0.13 (-0.30, 0.01)	-0.40 (-0.55, -0.26)	-0.27 (-0.40, -0.04)	-0.06 (-0.16, 0.04)	0.07 (-0.14, 0.27)
All Unplanned Rehospitalizations	All Hospital Discharges	Target	0.10 (-0.08, 0.29)	-0.29 (-0.44, -0.13)	-0.39 (-0.63, -0.15)	0.17 (0.07, 0.28)	0.07 (-0.14, 0.27)
		Non-Target	-0.04 (-0.22, 0.13)	-0.33 (-0.48, -0.18)	-0.29 (-0.53, 0.05)	0.03 (-0.07, 0.14)	0.07 (-0.14, 0.28)

Note: Slopes expressed as mean percentage point change per year in readmission rate for each policy period. Bolded values indicate within-group changes that are significantly different than baseline.

© 2022 Sabbatini AK et al. *JAMA Network Open.*

eTable 4. Sensitivity analysis comparing differential changes in readmission rate over time among HRRP-exposed and HRRP-exempt hospitals, under scenarios that exclude and include observation stays.

	Baseline (Period 1)	HRRP Announced (Period 2)	Δ (Period 2-Baseline)	HRRP Penalties Implemented (Period 3)	Δ (Period 3-Baseline)
Base Scenario (Inpatient Readmissions Following Inpatient Discharges Only)					
HRRP Hospitals	22.63% (22.40, 22.87)	21.98% (21.75, 22.20)	-0.66 (-0.98, -0.33)	20.39% (20.19, 20.59)	-2.24 (-2.55, -1.93)
Exempt Hospitals	22.37% (22.13, 22.60)	21.47% (21.24, 21.69)	-0.90 (-1.23, -0.57)	19.63% (19.43, 19.83)	-2.73 (-3.04, -2.42)
	Difference-in-Differences		*0.24 (-0.22, 0.70)*		*0.49 (0.05, 0.93)*
Expanded Scenario (Observation Stays Counted as Index Discharges and Readmission Events)					
HRRP Hospitals	23.86% (23.63, 24.09)	23.52% (23.30, 23.74)	-0.33 (-0.66, -0.02)	22.50% (22.31, 22.69)	-1.36 (-1.66, -1.06)
Exempt Hospitals	23.52% (23.29, 23.75)	22.94 % (22.72, 23.16)	-0.58 (-0.89, -0.26)	21.66% (21.46, 21.85)	-1.87 (-2.17, -1.56)
	Difference-in-Differences		*0.24 (-0.21, 0.69)*		*0.50 (0.08, 0.93)*

© 2022 Sabbatini AK et al. *JAMA Network Open.*
